# Silver Foams with Hierarchical Porous Structures: From Manufacturing to Antibacterial Activity

**DOI:** 10.1021/acsami.1c06057

**Published:** 2021-07-22

**Authors:** Fatma Cagla Durmus, José Miguel Molina Jordá

**Affiliations:** † University Materials Institute of Alicante, University of Alicante, Ap. 99, E-03690 Alicante, Spain; ‡ Inorganic Chemistry Department, University of Alicante, Ap. 99, E-03690 Alicante, Spain

**Keywords:** silver, silver−aluminum alloy, foam, hierarchical porous structure, dealloying, antibacterial

## Abstract

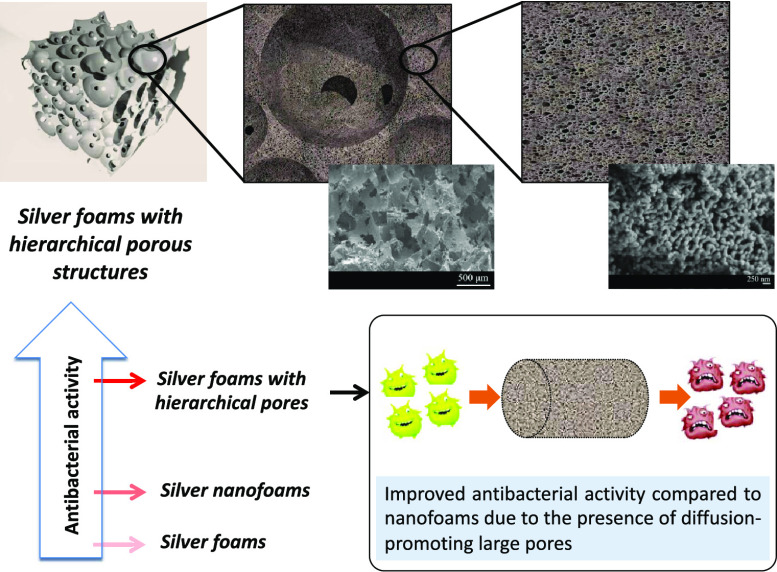

The development of porous materials with hierarchical porous structures is currently of great interest. These materials exhibit properties representative of different pore scales and thus open up the possibility of being used in new applications. In this paper, a method for the preparation of silver foams with hierarchical porous structures is discussed. Here, the replication method, which is typically used to produce coarse-pore foams, is merged with dealloying, which is commonly used to manufacture small-pore foams. For this purpose, packed NaCl particles (hard template) were infiltrated with 75%Al–25%Ag alloy (whose so-called soft template is the Al-rich phase). Both the hard and soft templates were removed by water dissolution and dealloying with HCl or NaOH solutions, respectively. Extensive characterization of the resulting materials revealed pores ranging from a few nanometers to hundreds of micrometers. The materials were characterized by their antibacterial performance against Gram-positive and Gram-negative bacteria and showed significantly higher activity than both silver foams prepared by sintering pure Ag particles and silver nanofoams produced by chemical dealloying. The combinations of pores of different sizes and the resulting high internal specific surface area have a decisive influence on the antibacterial capacity of these new materials.

## 1 Introduction

In recent years, there has been growing interest in the development of porous materials with specific pore sizes for applications as diverse as impact absorbents, filtration systems, thermal management systems, prosthetic implants, and catalysts.^1^ Each of these applications is optimized for a particular pore size.^2^ Porous materials with large pore sizes, ranging from micropores to macropores, are of interest when the pores are interconnected and fluids can flow through them.^3,4^ These materials are referred to as interconnected pore foams and can be of different nature (metallic, ceramic, polymeric, or composite).^3,5^ Since the average pore sizes in these materials vary from a few micrometers to millimeters,^3^ technological applications are mainly limited to their use as fluid particle filters or coolants in electronic systems. However, in the last decades, intensive efforts have been made to produce porous materials with pores in the nanometer range, characterized by a high specific surface area.^6^ These materials have been considered for various applications including electrodes, catalysts, sensors, actuators, and filtration systems.^3^ Activated carbon,^7^ activated alumina,^8^ or zeolites^6^ have been shown to be effective in the chemical catalysis of certain reactions, either by directly participating in the reaction mechanism or by supporting other catalyst materials.^6,7^ More recently, foams of various types (among which the metallic and ceramic foams stand out) with interconnected nanometer pores have been prepared by spinodal decomposition followed by selective dissolution of one of the phases present.^9^ In addition to the inherent advantages of high specific surface area, small-pore materials have distinct disadvantages due to restricted diffusion of species into and out of the pores, especially at large sample dimensions.^10^ This effect is particularly important for the innermost pores of a material, which often become nonfunctional due to access difficulties of diffusing species.^10^

Materials with hierarchical porous structures have had a significant impact on the scientific literature because they offer a combination of properties characteristic of different pore scales. In this scenario, the hierarchical pore size scales largely determine the intended applications for each material.^11^ This has led to materials with hierarchical porous structures at the nanometer scale, forming nanoarchitectures that are often used to control diffusing species.^12^ Other materials developed a hierarchical combination of pores at the macro/micro- and nanometer scales with specific applications in catalytic processes.^13^ An exciting application of certain porous metallic materials is their ability to eliminate bacteria.^14^ Metal ions such as copper, zinc, or silver have been shown to have potent antibacterial activity.^15^ Silver ions have a broad antibacterial spectrum as they negatively affect the growth of Gram-positive and Gram-negative bacteria. This is due to their ability to form ligand complexes with proteins or enzymes in bacterial cells.^16,17^ In recent work,^14^ the effect of micro/macroporous silver foams on the growth of certain bacteria was investigated. The foam to be used as an antibacterial material is expected to have a broad antibacterial spectrum, excellent mechanical resistance, and long-term biocidal activity. In addition, essential conditions must be considered, such as that it is not harmful to the environment and human health and does not cause toxic effects.^18^

The primary objective of this study is to develop effective antibacterial foams with hierarchically arranged pores ranging from millimeters to nanometers. The underlying principle is to merge pores of different sizes into a material with a large surface area (thanks to smaller pores), which in turn allows efficient molecular transport (which requires larger pores). The process used to produce these materials is a combination of the replication method, typically used to produce large-pore foams, and the selective dissolution method, generally used to manufacture small-pore foams. Production began by alloying aluminum and silver metals in a 75:25 ratio, and the resulting alloy was then used for gas pressure infiltration of packed NaCl particle preforms. NaCl was chosen as the hard template, while the aluminum in the alloy served as the soft template. After detailed structural characterization, experiments were performed to evaluate the foams for their antibacterial properties against Gram-positive and Gram-negative bacteria. These evaluations served to demonstrate the different roles of pores at different size scales in the elimination of bacteria and to explain the reason for the excellent antibacterial behavior of the foams with hierarchical porous structures prepared here.

## 2 Experimental Procedures

### 2.1 Materials

The materials used for the preparation of the alloy Al–Ag were high-purity 99.999% aluminum and 99.99% pure silver cast grain purchased from Alfa Aesar (GmbH & Co KG, Karlsruhe, Germany). NaCl particles with 99% purity were purchased from Panreac (Milan, Italy). These initial particles were sieved, and the fraction with an average diameter of 350–600 μm was collected. Analytically pure HCl and NaOH, purchased from Alfa Aesar (GmbH & Co KG, Karlsruhe, Germany), were used as selective dissolution reagents.

### 2.2 Preparation of Al–Ag Alloy

The Ag–Al alloy with atomic composition of 75%Al–25%Ag (hereafter referred to as Al–25Ag) was prepared in an induction furnace with up to 15 kW maximum power. For this purpose, appropriate amounts of the two metals were placed in a graphite crucible coated with boron nitride (BN). The crucible was heated to 1100 °C, and the liquid alloy was poured into a large graphite-coated steel mold after a waiting time of 3 min. The waiting time and the liquid metal movement generated by induction ensure the homogeneity of the sample composition. Casting into the steel mold allowed the metal to cool at about 300 K min^–1^ so that the sample could acquire a spinodal structure. The prepared metal with cylindrical dimensions of 15 mm diameter and 15 cm length was cut into smaller pieces of 5 cm height for use in infiltration experiments.

### 2.3 Fabrication of Ag Foams with Hierarchical Porous Structures

Ag foams with hierarchical porous structures were prepared by the following three-step method: (i) packing NaCl particles to conform packed particulate preforms and infiltrating such preforms with liquid Al–25Ag alloy, (ii) removing the NaCl templating agent by water dissolution to form Al–25Ag foams (hereafter referred to as AlAg), and (iii) dissolving the Al-rich phase by a chemical attack with aqueous solutions of HCl or NaOH to form the final Ag foam. Each step is explained in detail below.

The NaCl particles were packed into cylindrical BN-coated graphite crucibles with a height of 30 mm and a diameter of 18 mm. A method developed by the Alicante University group was used, which involved alternating vibrations and impacts applied with a metal plunger. The attained volume fraction was 0.58 ± 0.01, which is slightly lower than the value encountered in the literature for random packing of spherical particles (in the range 0.59–0.64, depending on the packing conditions). A piece of Al–25Ag alloy was placed over the packed preform, and the assembly was placed in an infiltration chamber. The infiltration chamber consists of a metal chamber that can be pressurized to a maximum pressure of 0.45 MPa and is equipped with an electric resistance furnace that enables metal melting. The vacuum was set to 0.1 mbar. At the same time, the temperature was increased to 1033 K at 5 K min^–1^. After another 5 min, necessary to ensure temperature homogenization, the vacuum was stopped and a pressure of 0.1 MPa was applied to the chamber. This pressure was sufficient to infiltrate the liquid Al–25Ag alloy into the porous preform. Then, the crucible and sample set were directionally cooled by moving them to the lower part of the chamber, where they fit into a water-cooled copper cooler. The resulting solidification rate (up to 5 K s^–1^) is nearly equivalent to what can be achieved by pouring molten metal into a metal mold. Once the metal had solidified, the sample was extracted and the excess metal was removed.

**Figure 1:**
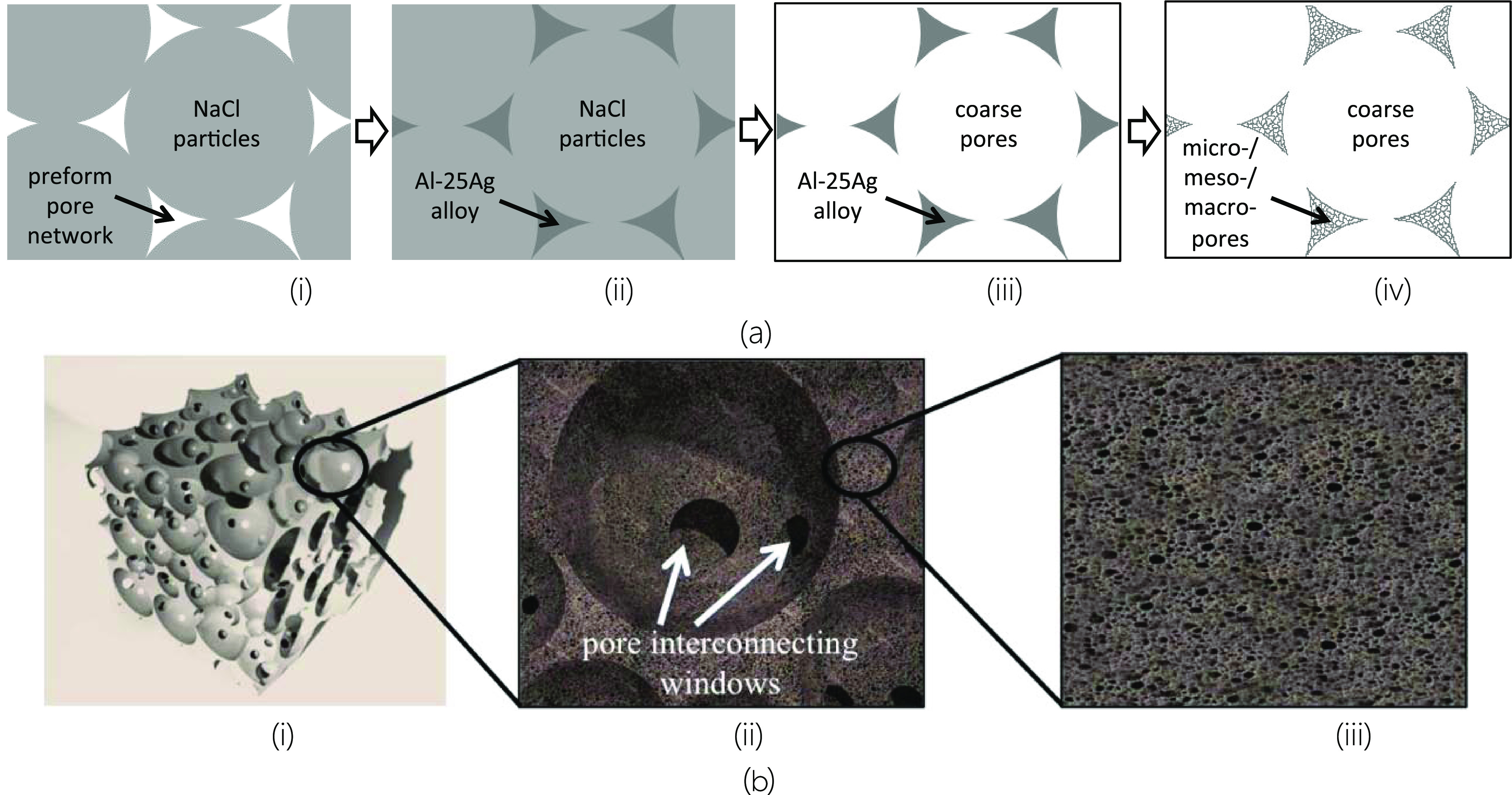
(a) Schematic representation of the main steps in the production of Ag-based foams with hierarchical pore structures: (i) packing NaCl particles (hard template) to obtain a porous preform, (ii) infiltrating the porous preform in (i) with Al–25Ag, (iii) dissolving the NaCl particles in water to generate a foam structure with coarse pores, and (iv) selectively dissolving in acidic or alkaline media to generate micro/meso/macropores in the initial foam struts in (iii). (b) Various three-dimensional zoom drawing views of hierarchical porous foams produced in the present work: (i) general view, (ii) zoomed view showing coarse pores and pore-connecting windows, and (iii) zoomed view showing micro/meso/macropores developed in struts.

The resulting material is a composite of Al–25Ag metal matrix and NaCl particles. Both the NaCl particles and the Al-rich phases in the matrix can be considered as templates that allow different types of porosities to be obtained after dissolution. NaCl particles are a rigid templating agent and can be removed by a two-step dissolution process (see refs^19, 20^ for details). The result is an AlAg foam with pores that replicate the characteristics of the NaCl particles. Subsequently, the Al-rich phase of Al–25Ag, which is considered a soft templating agent, is removed. For this purpose, the samples are treated with chemicals such as HCl or NaOH (both reagents have been used in the literature to effectively dissolve Al-rich phases in Al–Ag alloys). Acidic treatment was performed by immersing the Al–25Ag foam sample in a 5% HCl solution at 300 K for 8 h. The alkaline medium treatment consisted of immersing the sample in a 20% NaOH solution at 300 K for 8 h. The diagram in Figure 1 shows the different templating agents and their removal treatments.

### 2.4 Fabrication of Ag Foams by Particle Sintering

Sintered foams were produced by a simple sintering process consisting of two steps: (i) packing spherical pure silver particles (99.99%) with an average diameter of 0.7–1.3 mm into a quartz tube with a diameter of 16 mm and (ii) treatment at 873 K for 300 min in an electric furnace with an inert nitrogen flow atmosphere at 5 mL min^–1^.

### 2.5 Characterization of Ag Foams

#### 2.5.1 Microstructural and Chemical Featuring

The foam morphology was studied by scanning electron microscopy (SEM) using a Hitachi S3000N equipment and by field-emission scanning electron microscopy (FESEM) using a Zeiss Merlin VP equipment. For this purpose, SEM and FESEM images were acquired at different magnifications and analyzed using image analysis software Buehler-Omnimet Enterprise (Illinois). An X-ray Bruker XFlash 3001 EDX probe connected to the former electron microscope was used to study the chemical composition. A Bruker D8 Advance X-ray diffractometer was used to determine the crystalline phases in the metal during the various processing steps.

#### 2.5.2 Characterizing Fine Microstructure from Nitrogen Adsorption Isotherms

To obtain information about the pores in a size interval below 50 nm, an indirect study was performed by analyzing the nitrogen adsorption curves of the foam samples. These curves were recorded at 77 K using an Autosorb 6-b Quantachrome Instruments equipment (Florida). The isotherms were analyzed using two complementary theories. The first one is the well-known standard BET theory, which allows obtaining the specific sample surface area. In addition, the theoretical framework developed in ref (21) was used to obtain pore size distribution profiles in the range of 0–50 nm.

#### 2.5.3 Antibacterial Activity

Antibacterial tests for *Escherichia coli* and *Staphylococcus aureus* were performed according to ASTM E2149-13a.^22^ Bacterial population growth curves of both bacteria were generated in Luria broth (LB) media before testing antibacterial activity. A colony from a single colony plantation was seeded in a nutrient broth and incubated at 37 °C for 24 h. An aliquot of this culture was transferred to a 250 mL Erlenmeyer flask containing 50 mL of liquid medium. Incubation was carried out in an orbital shaker incubator at 310 K and 200 rpm, and the optical density was measured. The reason for choosing 310 K as the incubation condition for bacteria is that at this temperature both bacteria can divide and multiply optimally. Based on the growth curves of the bacterial population, the maximum growth time of the bacterial population was determined. These times were set at 5.5 h for *E. coli* and 6 h for *S. aureus* using a known concentration of the bacterial inoculum. The inoculum of *E. coli* and *S. aureus* was prepared using a total volume of 9 mL of liquid culture medium containing 1.70 × 10^5^ and 1.81 × 10^5^ CFU mL^–1^, respectively (here, we use the terminology commonly used in microbiology of colony-forming unit or CFU, which is defined as a unit for estimating the number of viable bacteria or fungal cells in a sample; viable means microorganisms that can multiply by binary fission under controlled conditions). Samples were placed in the prepared liquid medium and incubated at 310 K with a rotation of 200 rpm. The bacterial concentrations were determined at different time intervals (4, 6, 8, 10, and 24 h), and the values of percentage reduction and logarithmic reduction were calculated.

## 3 Results and Discussion

### 3.1 Manufactured Specimens

**Figure 2:**
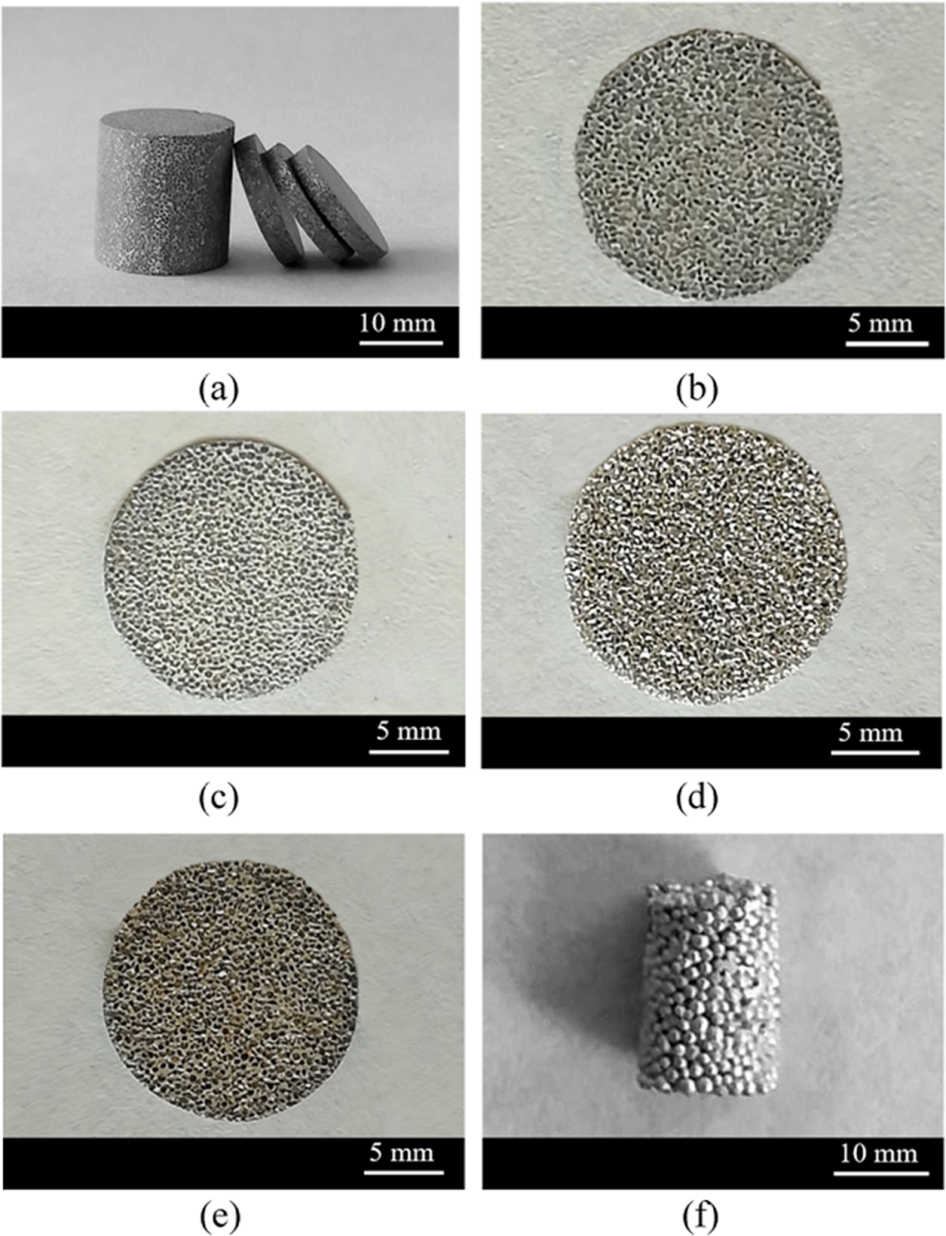
Photographs of the manufactured specimens. (a, b) General view of the composite material obtained by infiltrating packed NaCl particle preforms with Al–25Ag alloy. (c–f) Foams corresponding to the following sample codes: (c) AlAg, (d) Ag–HCl, (e) Ag–NaOH, and (f) Ag-sint.

Table 1 displays the codes of the different foams prepared in this work and a reminder of the pore formation treatments. The samples coded as AlAg, Ag–HCl, and Ag–NaOH appear to be equivalent (Figure 2), except for a slight color difference, as the Ag–HCl sample has a slightly yellowish tonality and the Ag–NaOH sample is darker than the others. At first sight, there is no difference in porosity between the samples, as the higher porosities of the Ag–HCl and Ag–NaOH samples are only noticed after measuring their densities (Table 2), which are lower (1.23 and 1.41 g cm^–3^, respectively) than that of the AlAg sample (1.87 g cm^–3^). The Ag-sint sample has a distinct appearance as it consists of sintered spherical particles with a size of 0.7–1.3 mm, leaving pores of similar size due to their low sintering degree. Its density (6.55 g cm^–3^) is significantly higher than that of the other materials, as Ag has high density and is also present in greater quantity (the Ag spheres are densely packed up to a volume fraction of 0.63, far more than the metal volume fraction achieved in the AlAg material, which is 1 – 0.58 = 0.42; 0.58 is the NaCl particle volume fraction). The metal volume fraction in the Ag–HCl and Ag–NaOH samples is much lower because some of the metal is dissolved by the chemical treatment with HCl and NaOH (the selective chemical dissolution increases the initial porosity resulting from the NaCl particles by 52 and 47% in the Ag–HCl and Ag–NaOH samples, respectively).

**Table 1:** Sample Codes of Manufactured Samples and the Pore Formation Treatments

sample code	material	coarsest pore generation procedure	smallest pore generation procedure
Al–25Ag	Al–25Ag alloy	none	none
AlAg	Al–25Ag foam	water dissolution of NaCl	none
Ag–HCl	Ag foam	water dissolution of NaCl	chemical treatment with HCl
Ag–NaOH	Ag foam	water dissolution of NaCl	chemical treatment with NaOH
Ag-sint	Ag foam	sintering of Ag particles	none

**Table 2:** Total Pore Volume Fraction *V*_p_ (in cm^3^ Pores per cm^3^ Material) and Specific Pore Volume (in cm^3^ Pores per g Material) of the Ag–Al Alloy and Different Foams, Calculated from Densities Obtained by Densitometry (ρ_app_ or Apparent Density, in g cm^–3^) and Helium Picnometry (ρ_He_, in g cm^–3^)

sample code	ρ_app_ (±0.03)	ρ_He_ (±0.03)	pore volume fraction (*V*_p_ ± 0.01)	specific pore volume (±0.01)
Al–25Ag	4.38	4.37	≈0.0	≈0.0
AlAg	1.87	4.36	0.58	0.32
Ag–HCl	1.23	10.2	0.88	0.72
Ag–NaOH	1.41	9.72	0.86	0.61
Ag-sint	6.55	10.38	0.37	0.06

### 3.2 Phase Constitution and Chemical Composition

**Figure 3:**
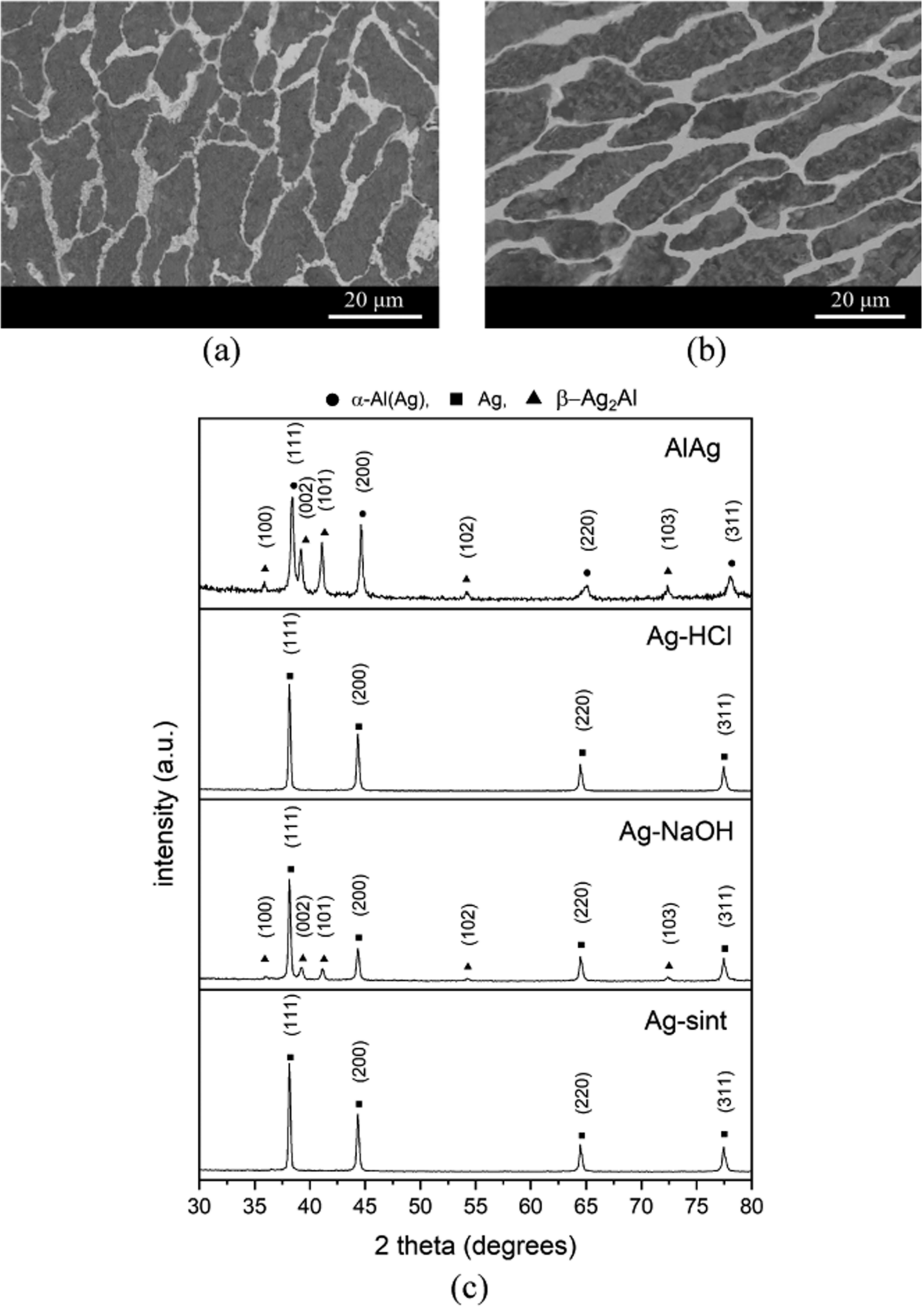
(a, b) Optical microscopy images of Al–25Ag alloy under two metallurgical conditions: (a) after rapid solidification by casting process and (b) after infiltration. (c) XRD patterns corresponding to different samples—from top to bottom: AlAg, Ag–HCl, Ag–NaOH, and Ag-sint.

Figure 3a shows an optical micrograph of the Al–25Ag alloy in the as-cast condition. According to quantitative image analysis, two phases can be distinguished: a darker and a lighter phase in a ratio of 73 and 27%, respectively. A thorough EDX examination of the two phases indicates that the dark phase is rich in Al, with a silver content of about 9.34 at. %, while the light phase has an atomic Ag/Al ratio of 2:1 (Table 3). Figure 3b displays a similar micrograph depicting the metallurgical state of the Al–25Ag alloy after the infiltration process (the micrograph was taken in the interparticle region). The percentages of dark and light phases measured by image analysis are 72 and 28%, respectively. There are no significant differences between the two micrographs shown in Figure 3a,b, except that the crystals corresponding to the dark phase of the infiltrated alloy are more homogeneous and larger. This is likely due to the castlike solidification conditions used and the role of NaCl particles in promoting nucleation during metal solidification. Analysis of these phases by EDX gives similar results to the castlike alloy with a slightly lower average Ag content of 7.86% in the dark phase.

**Table 3:** Chemical Composition (at. %) of the Manufactured Samples Measured by EDX Analysis

	chemical elements				
sample code		Ag	Al	O	C
Al–25Ag alloy	global	24.6	73.8	1.20	0.36
	dark phase^a^	9.34	89.0	1.27	0.41
	light phase^a^	65.8	32.9	1.01	0.23
	dark phase^b^	7.86	90.4	1.35	0.34
	light phase^b^	65.1	33.7	0.83	0.41
AlAg		24.7	74.2	0.88	0.25
Ag–HCl		98.5	0.69	0.54	0.28
Ag–NaOH		88.9	9.01	1.72	0.36
Ag-sint		99.8	0.00	0.15	0.10

a As cast.

b After infiltration.

Figure 3c displays the Al–25Ag XRD spectrum in which the peaks corresponding to the α-Al(Ag) and β-Ag_2_Al phases can be identified. These peaks correspond respectively to the dark and light phases previously characterized by microscopy and EDX. Therefore, the concentration of Ag in the dark phase measured by EDX (9.34 and 7.86 at. % for the as-cast and postinfiltration conditions, respectively) thus corresponds to silver dissolved in a primary aluminum crystal matrix. The equilibrium phase diagram of the system Al–Ag indicates that the α-Al(Ag) and Ag_2_Al phases can coexist at room temperature and that the maximum silver solubility in the α-Al(Ag) phase at the eutectic temperature is 23.5 at. %. A wide range of percentages of silver dissolved in the α-phase has been reported in the literature for the Al–Ag system based mainly on solidification conditions: from 2.2 at. %^23^ for relatively slow solidification conditions to 23.5 at. % for a supersaturated α-phase obtained by ultrafast cooling.^24^ The lower value of dissolved silver in the primary α-Al(Ag) phase (dark) of the Al–25Ag alloy after infiltration (7.86 at. % compared to 9.34 at. % recorded for the as-cast condition) can then be explained by milder solidification conditions. The EDX results allow calculating that the percentage of phases in the Al–25Ag alloy after infiltration is about 71% for α-Al(Ag) and 29% for the β-Ag_2_Al phase, which is in perfect agreement with the results from image analysis by optical microscopy.

Figure 3c also shows the XRD spectra of the Ag–HCl, Ag–NaOH, and Ag-sint samples. Overall, these XRD patterns are consistent with the previously published literature on Al–Ag alloys with similar Ag contents treated with HCl and NaOH.^23,25,26^ Analysis of the XRD pattern for the Ag–HCl sample shows not only that the α-Al(Ag) phase was completely dissolved but also that the Al contained in the intermetallic β-Ag_2_Al was dissolved concurrently. It was shown that dissolution of the primary α-Al(Ag) crystals in HCl acid creates penetration pathways that exhibit a catalytic effect for the dissolution of β-Ag_2_Al in biphasic Al–Ag alloys. Thereby, the metal matrix after acid treatment consists of a single Ag phase with the FCC structure. EDX analysis of the Ag–HCl sample confirms a pure Ag composition with impurities of C, Al, and O (Table 3). Dealloying in NaOH leads to different results, as peaks corresponding to pure silver and others associated with the remaining intermetallic β-Ag_2_Al are found in the XRD spectrum of the Ag–NaOH sample. This is evidence that NaOH is not able to completely eliminate this intermetallic phase, as observed in previous work.^27^ The EDX analysis reveals high Al presence, which is in agreement with the XRD results. Finally, the XRD and EDX analyses show only Ag for the Ag-sint sample.

### 3.3 Morphology and Size Distribution of the Coarse Pores

**Figure 4:**
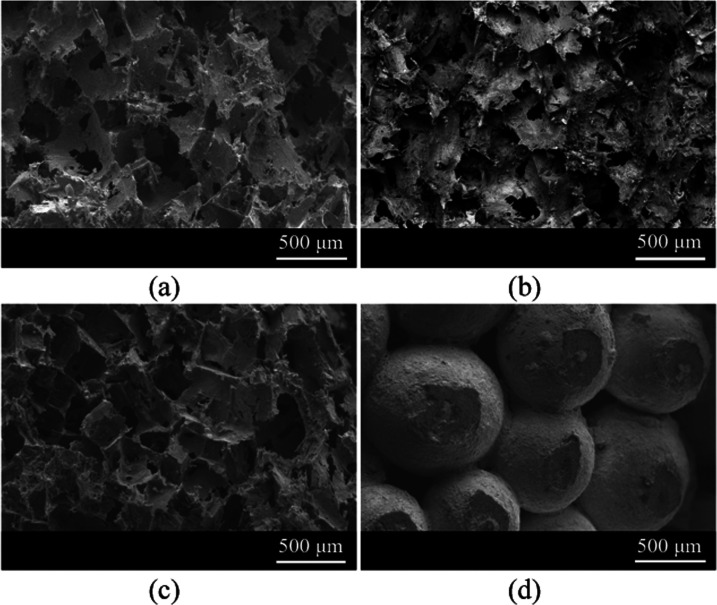
Scanning electron microscopy (SEM) images of the following samples: (a) AlAg, (b) Ag–HCl, (c) Ag–NaOH, and (d) Ag-sint.

Figure 4 illustrates the morphology of the largest (coarse) pores in each sample. No obvious differences are observed in the AlAg, Ag–HCl, and Ag–NaOH samples (micrographs a, b, and c, respectively). In all three cases, a structure of interconnected pores is observed. The pores are the size of the original NaCl particles and establish interconnections through micrometer-sized windows. In contrast, the Ag-sint sample (Figure 4d) exhibits a special microstructure developed by sintered Ag spheres and has an inverse pore structure compared to the above. Again, the pores are interconnected, but their characteristic dimension is determined by the degree of sintering.

**Figure 5:**
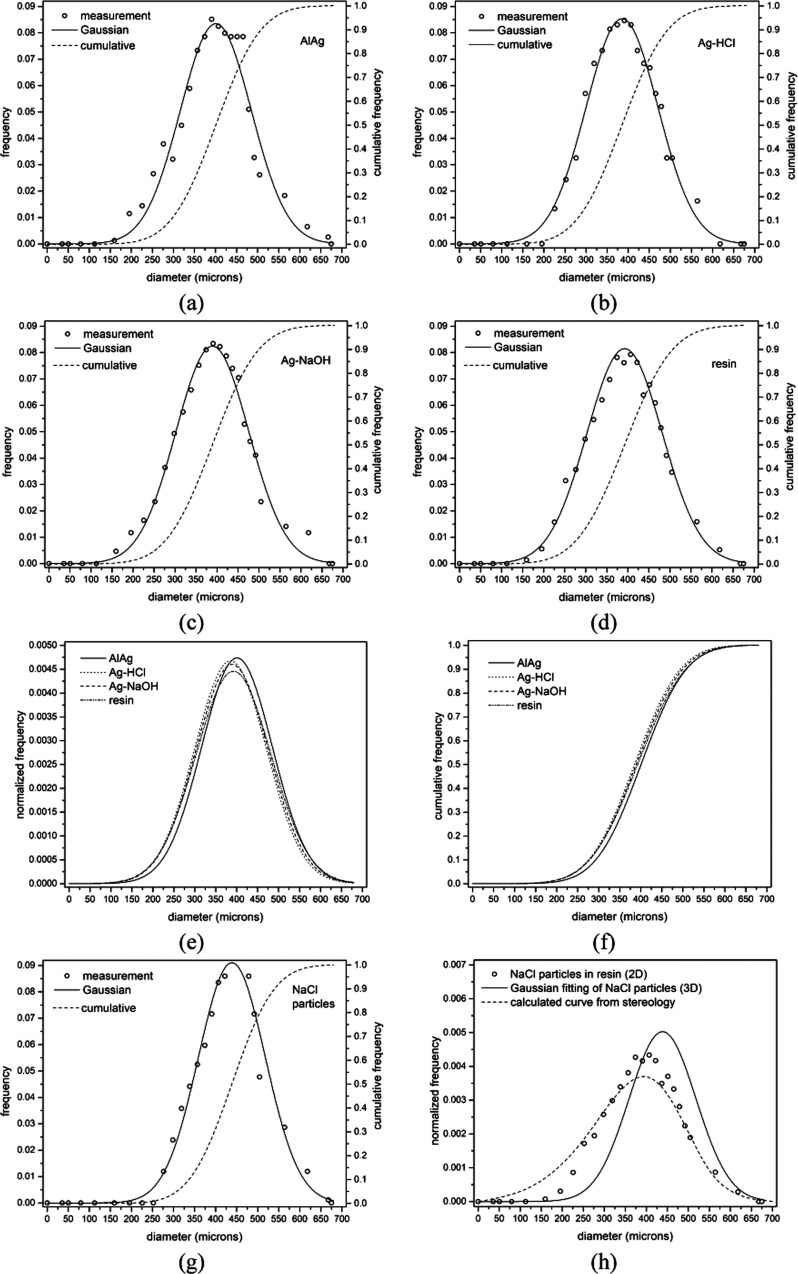
Experimental pore size distribution frequency data, Gaussian function fitting curves, and cumulative pore size distribution curves of the coarse pores for (a) AlAg, (b) Ag–HCl, (c) Ag–NaOH, and (d) a sample prepared by cutting and polishing (2D measurements) NaCl particles embedded in resin. (e, f) Compilations of normalized and cumulative frequency curves, respectively, of the above samples. (g) Pore size distributions of NaCl particles (3D measurements). (h) Comparison of the normalized frequencies of (i) NaCl particles embedded in resin (2D measurements) and (ii) NaCl particles (3D measurements) and (iii) 3D curve calculated from the 2D curve using stereology.

Figure 5a–c shows the size distributions of the coarse pores (pore diameter > 2 μm) in AlAg, Ag–HCl, and Ag–NaOH samples. These measurements were collected by analyzing each sample on a cut and polished surface. Additionally, Figure 5d shows the results for a sample with NaCl particles embedded in resin. Each figure shows experimental data on the frequency size distribution (obtained by image analysis), the fit of the curve to a Gaussian function, and the cumulative size distribution.

Previously, for all samples in Figure 5, it was verified that Weibull size distributions, commonly used for particles (or the porous cavities they originate from) produced by grinding and sieving, give similar results to Gaussian distributions. Figure 5e,f displays all curves defined by their normalized frequencies (sum equal to one for each sample of all observable frequencies) and their cumulative frequencies, respectively. In both frequency plots, all curves overlap significantly, which explains the common origin of the coarse pores in all samples. The size distribution of NaCl particles embedded in resin (Figure 5d) allows the prediction of the size distribution of NaCl particles (considered as 3D objects and shown in Figure 5g) using the statistical stereological particle model described in ref (28). The model in ref (28) considers a homogeneous form distribution and polydisperse sizes. Figure 5h compares the results for NaCl particles embedded in resin, Gaussian fitting of NaCl particles, and the stereologically determined distribution. Calculated and measured distributions for NaCl particles are reasonably well correlated. From these close correlations, it can be concluded that the formation of the coarse pores (pore diameter > 2 μm) in these samples is solely due to water dissolution of the NaCl templating agent.

### 3.4 Morphology and Contribution to the Specific Surface Area of the Finest Pores

**Figure 6:**
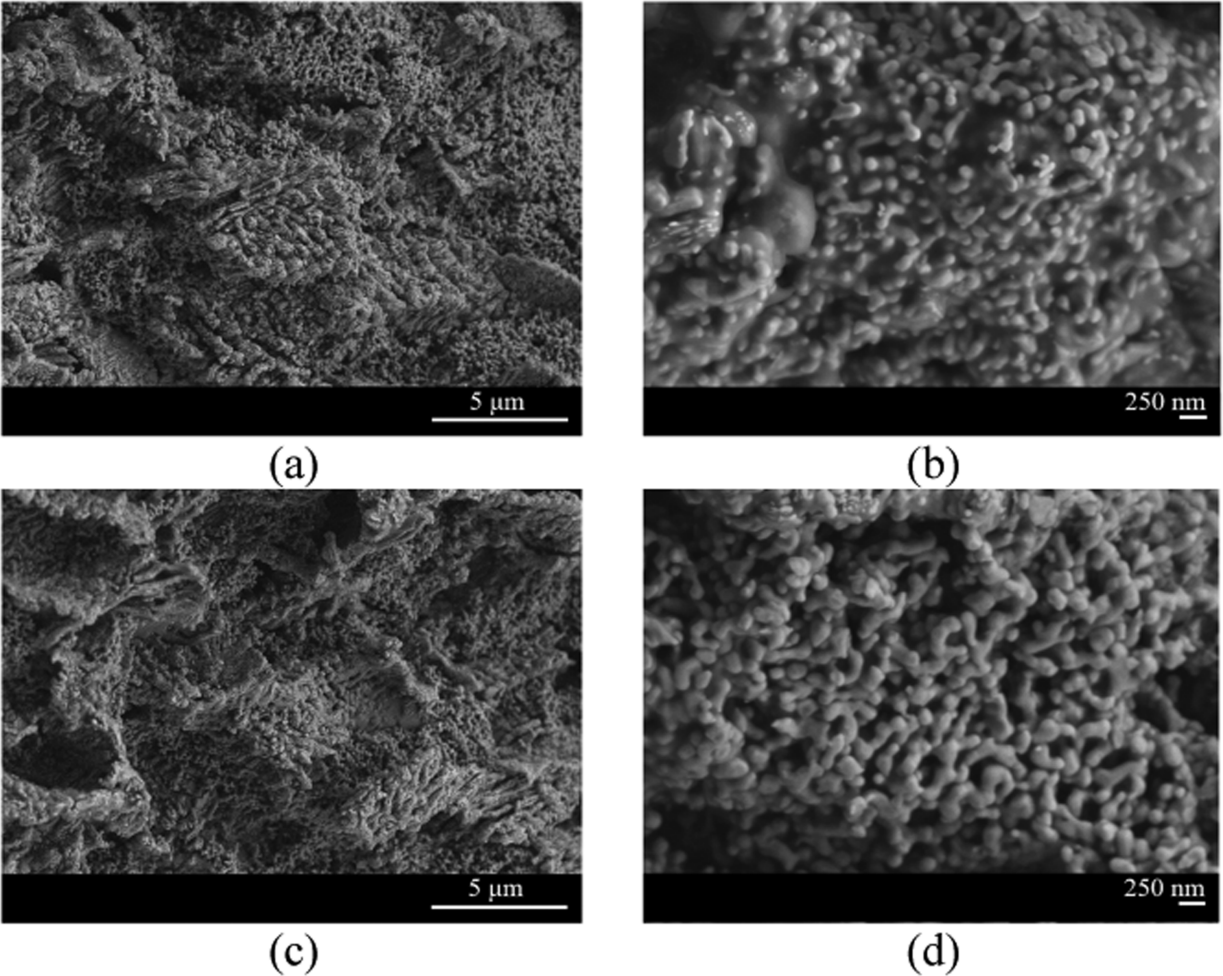
FESEM micrographs in different sample regions showing porosity generated by selective chemical dissolution with (a, b) HCl and (c, d) NaOH.

Fine porosity in foams was developed by selective dissolution in acidic (HCl) and alkaline (NaOH) media. Figure 6 shows the microstructures of Ag–HCl and Ag–NaOH samples in which homogeneous ligament patterns are observed. The porous structures produced by both treatments show significant similarities as both materials exhibit a 3D bicontinuous nanoporous structure. High-magnification micrographs (Figure 6b,d) show good mechanical integrity with no visible cracks.

Porous structures formed during the dealloying of alloys containing noble elements such as Ag are influenced by the surface diffusivity of the noble atoms^26,29,30^ and can be affected by the properties of the solutions such as temperature, ionic composition, and concentration. The less noble components are dissolved out of the precursor alloy, while the remaining noble component diffuses and agglomerates to form a nanoporous structure. Both the surface diffusion of Ag atoms in Al–Ag alloys and the reaction between Al and chemical etchant determine the nanoporous structure. The silver surface diffusion coefficient can be estimated by the following equation^26,29^
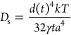
1where *k* is the Boltzmann constant (1.3806 × 10–23 J K^–1^), γ is the surface energy of Ag (1.24 J m^–2^), *d*(*t*) is the ligament size of the as-dealloyed Al–25Ag alloy at the dealloying time *t*, *a* is the lattice parameter of Ag (4.086 × 10^–10^ m), and *T* is the dealloying temperature. The ligaments developed in Figure 6 have homogeneous but different dimensions for acidic and alkaline treatments. The ligaments of acid-treated samples have a diameter of about 115–130 nm, but only about 65–80 nm for NaOH-treated samples. In ref (30), the authors used a Ag_25_Al_75_ alloy and obtained ligaments of different sizes by treating it with 5 wt % HCl at different temperatures and times. They concluded that the surface diffusion of Ag is an activated process according to the Arrhenius law with an activation energy of 75.66 kJ mol^–1^ and a pre-exponential factor *D*_o_ = 4.432 × 10^–4^ m^2^ s^–1^. Considering these values in the Arrhenius equation, we can deduce that the surface diffusion coefficient of Ag for the temperature used in this study (300 K) is *D*_s_ = 2.973 × 10^–17^ m^2^ s^–1^. Using eq 1, we can calculate from these data that the ligament size that develops during the treatment of Al–25Ag alloy with 5 wt % HCl for 28 800 s (8 h) at 300 K (conditions used in this study) must be about 123 nm, in full agreement with the experimental results of Figure 6 (about 115–130 nm).

The literature on dealloying of Al–Ag alloys in NaOH media is limited to a few studies.^24,25,27,31^ In ref (27), the authors concluded that the initial composition of the different precursor Al–Ag alloys and the composition of the dealloying solutions (HCl vs NaOH) have a significant effect on the development of nanoporosity in the final materials, with smaller ligament dimensions found for treatments with NaOH vs HCl. One of the most relevant studies is ref (25), which concluded that the presence of chlorides (Cl^–^) in the HCl solution can accelerate the surface diffusion of undissolved noble elements and thus induce ligament coarsening in the final nanoporous structures. The authors of ref (31) reached a similar conclusion, this time using an Al–32Cu alloy.

**Figure 7:**
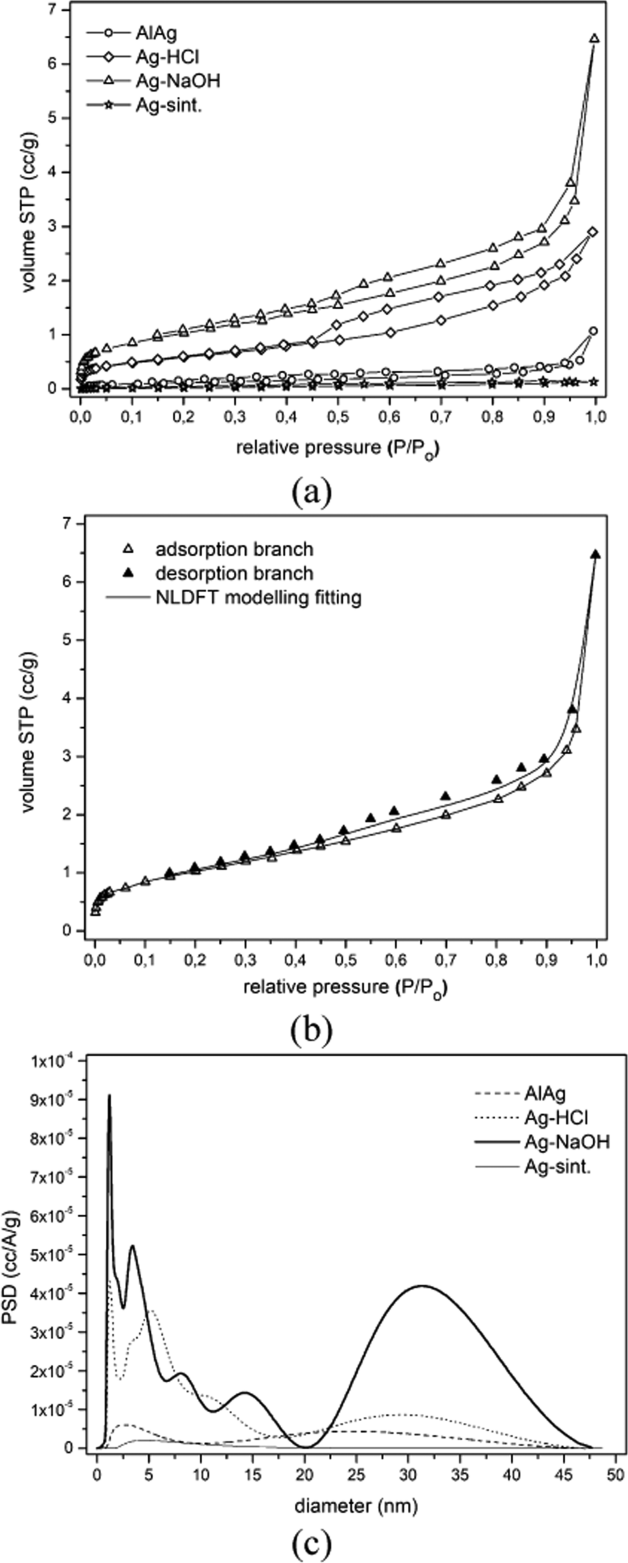
(a) Nitrogen adsorption/desorption isotherms at 77 K. (b) NLDFT model fit of the adsorption/desorption isotherm at 77 K of the Ag–NaOH sample. (c) Pore size distribution as a function of diameter calculated with the NLDFT model.

Figure 7a shows the nitrogen adsorption isotherms for the different samples. According to the IUPAC classification,^32^ these are isotherms with sufficient characteristics to be classified as type II. For the Ag–HCl and Ag–NaOH samples, the isotherms show a combination of a well-defined H3 hysteresis loop at high relative pressures in the range of 0.8–1.0 and an H2 hysteresis loop at relative pressures in the range of 0.2–0.8. The presence of the H3 hysteresis loop at high relative pressures and the absence of a plateau at *P*/*P*_0_ = 1.0 indicate a solid with a macropore size distribution. However, the H2 hysteresis loop indicates the presence of mesopores. Isotherms with similar characteristics have been obtained for hierarchical porous materials that have a combination of macropore and mesopore structures.^33,34^ It is observed that the adsorption and desorption branches of AlAg and Ag-sint samples overlap and no visible hysteresis behavior is found (even rescaling Figure 7a for a magnified view). This is a clear indication of the absence of mesopores in these materials, which can be assigned a mono-macro-porosity. These adsorption–desorption curves were studied in detail using BET theory to obtain the specific surface areas and volumes for each solid (Table 4).

**Table 4:** Specific Surface Area (*S*_BET_, in m^2^ g^–1^) and Distribution of Specific Pore Volumes (in cm^3^ g^–1^) for the Different Samples

sample code	*S*_BET_	*V*_total_	*V*_micro_	*V*_meso_
AlAg	0.425	305 × 10^–3^	1.74 × 10^–4^	4.62 × 10^–4^
Ag–HCl	2.10	715 × 10^–3^	7.56 × 10^–4^	3.73 × 10^–3^
Ag–NaOH	4.08	606 × 10^–3^	1.31 × 10^–3^	8.69 × 10^–3^
Ag-sint	0.215	560 × 10^–4^	5.05 × 10^–5^	1.33 × 10^–4^

The Ag–NaOH sample shows the highest BET surface area (4.08 m^2^ g^–1^), followed by Ag–HCl (2.10 m^2^ g^–1^). These materials show comparable total pore volume but differ significantly in the distribution of finer pores. As previously reported in the literature, the surface diffusivity of Ag during alkaline solution dealloying of Al-based alloys is significantly lower than in acidic medium due to the formation of MN-hydroxy (MN, more noble) compounds. Therefore, the coarsening ability of silver atoms is much lower and larger amounts of smaller pores were obtained compared to the acidic medium.^27,31^ The AlAg and Ag-sint samples show low specific surface areas (0.425 and 0.215 m^2^ g^–1^, respectively) due to the absence of microporous or mesoporous structures. The specific surface area of NaCl particles is 0.740 m^2^ g^–1^, which corresponds to 1.60 m^2^ cm^–3^ (the density of NaCl was assumed to be 2.16 g cm^–3^). Provided that these particles are packed to a volume fraction of 0.58, the specific surface area of a packed preform made of these NaCl particles is approximately 0.928 m^2^ cm^–3^, which corresponds to 0.496 m^2^ g^–1^ for AlAg foam (the density of AlAg foam was taken as 1.87 g cm^–3^ from Table 2). This value is slightly higher than the value measured for the AlAg sample. This is certainly due to the fact that the manufacturing process replicates the characteristics of NaCl particles except for the interparticle contact regions. Those areas remain uninfiltrated and form connecting windows between the pores. As these uninfiltrated regions decrease with increasing infiltration pressure, the specific surface areas of the NaCl particles and their derived foams are expected to gradually correlate at high infiltration pressures.

The NLDFT model was used to determine the cumulative pore size distribution in the different samples. Although this model was not specifically developed for metallic solids, it has been successfully applied to nanoporous silver structures.^35^Figure 7b shows an acceptable overlap between the experimental and calculated isotherms from a model framework created for mesostructured carbons. The size distribution plots shown in Figure 7c confirm the assertions made so far: Ag–HCl and Ag–NaOH samples show mesoporosity, while AlAg and Ag-sint display no signs of mesoporosity.

### 3.5 Antibacterial Activity

Table 5 provides information on the antibacterial characterization of the samples. The terms “percentage reduction” and “log (reduction)” are commonly used in microbiology and antimicrobial testing (antimicrobial assays), as defined below^22^
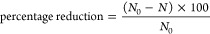
2
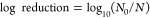
3where *N*_0_ is the number of viable microorganisms before treatment and *N* is the number of viable microorganisms after treatment.

**Table 5:** Concentration of Bacteria (in CFU mL^–1^) over Time (*t*, in h) in Media with the Presence of the Foams Produced in This Work

	*E. coli* concentration (CFU mL^–1^)				*S. aureus* concentration (CFU mL^–1^)			
*t*	AlAg	Ag–HCl	Ag–NaOH	Ag-sint	AlAg	Ag–HCl	Ag–NaOH	Ag-sint
0	1.70 × 10^5^	1.70 × 10^5^	1.70 × 10^5^	1.70 × 10^5^	1.81 × 10^5^	1.81 × 10^5^	1.81 × 10^5^	1.81 × 10^5^
1	1.40 × 10^5^	1.05 × 10^5^	6.98 × 10^4^	1.61 × 10^5^	1.60 × 10^5^	1.20 × 10^5^	8.99 × 10^4^	1.66 × 10^5^
2	1.32 × 10^5^	3.83 × 10^4^	2.60 × 10^4^	1.49 × 10^5^	1.44 × 10^5^	4.00 × 10^4^	2.03 × 10^4^	1.53 × 10^5^
4	1.00 × 10^5^	2.80 × 10^4^	9.07 × 10^3^	1.38 × 10^5^	1.21 × 10^5^	6.30 × 10^3^	3.23 × 10^3^	1.31 × 10^5^
6	7.83 × 10^4^	1.00 × 10^4^	2.98 × 10^3^	1.16 × 10^5^	8.80 × 10^4^	2.84 × 10^3^	7.31 × 10^2^	1.10 × 10^5^
8	5.00 × 10^4^	6.32 × 10^3^	7.50 × 10^2^	1.01 × 10^5^	6.12 × 10^4^	1.92 × 10^3^	2.96 × 10^2^	1.00 × 10^5^
10	4.08 × 10^4^	4.56 × 10^3^	2.51 × 10^2^	9.10 × 10^4^	3.80 × 10^4^	1.21 × 10^3^	1.55 × 10^2^	8.52 × 10^4^
16	2.05 × 10^4^	1.51 × 10^3^	5.03 × 10^1^	5.04 × 10^4^	1.78 × 10^4^	6.20 × 10^2^	3.70 × 10^1^	5.50 × 10^4^
24	5.01 × 10^3^	1.40 × 10^2^	5.50 × 10^0^	1.29 × 10^4^	1.11 × 10^4^	2.48 × 10^2^	1.15 × 10^1^	2.91 × 10^4^

**Figure 8:**
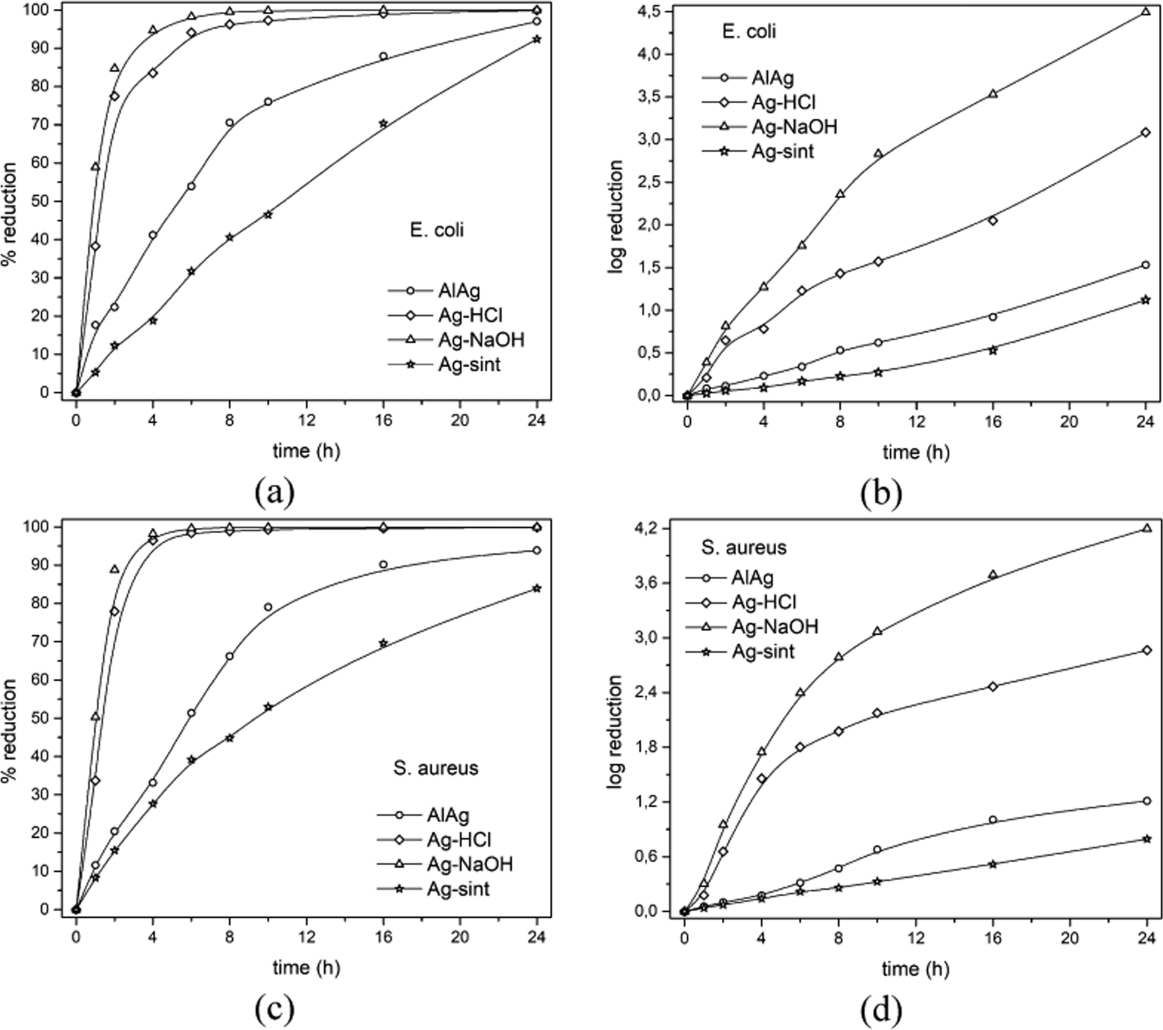
Results of antibacterial tests for (a, b) *E. coli* and (c, d) *S. aureus*, shown as percentage reduction as a function of exposure time (a, c) and as logarithmic reduction as a function of exposure time (b, d).

Figure 8a,c presents the data from Table 5 showing the percentage reduction of bacteria over time. It can be seen that there is no significant difference in bactericidal activity for the two types of bacteria, although there is a slight time lag in the reduction of *S. aureus* bacteria. This could be due to the fact that *S. aureus* has a thicker cell membrane than *E. coli* and usually has a higher bactericidal resistance.^36,37^ Regarding the specific activity of each material, the bactericidal activity of the Ag–NaOH sample stands out with a percentage reduction of 99 within about 5 h for *S. aureus* and 7 h for *E. coli*. The Ag–HCl sample shows fairly strong bactericidal activity but less than that of Ag–NaOH, as it takes about 9 h for *S. aureus* and 16 h for *E. coli* to reach 99% reduction. In the 24 h analysis, the bactericidal activity of both materials, Ag–NaOH and Ag–HCl, is far superior to that of AlAg and Ag-sint, which achieve low percentage reductions of 97 and 92 for *E. coli* (94 and 84 for *S. aureus*), respectively. In terms of log(reduction), Figure 8b,d shows the bactericidal capacity as log-reduction performances of all materials. Note that Ag–NaOH reaches log reductions of 4.5 and 4.2, while the maximum values for the other materials are 3.1 and 2.9 for Ag–HCl, 1.5 and 1.2 for AlAg, and 1.1 and 0.8 for Ag-sint (the values for each material are given for *E. coli* and *S. aureus*, respectively). It should be recalled that substances and materials are deemed antimicrobial if they can achieve a 2-log or greater reduction in colony-forming organisms.^38^ Nevertheless, there are no internationally accepted methods for antimicrobial testing except ISO 22196:2011, ASTM 2149-13a, and ASTM E2180-18.^38^ In recent years, numerous articles have been published on silver-based products and materials with bactericidal activity, including silver nanoparticles^39−42^ and silver ion-releasing materials.^39,43^ Studies with both materials indicate greater bactericidal activity than the materials prepared here. By adding 0.2 ppm Ag^+^ ions to the culture media, log-reduction values of 6 can be achieved for *S. aureus* after 3 h and for *E. coli* after 1 h.^38^ The use of silver nanoparticles or silver derivatives generally leads to a significant decrease in activity compared to the direct use of Ag^+^ ions. In fact, metallic (nonionic, Ag^0^) silver has no antibacterial effect.

The activity of silver relies on the fact that Ag^+^ ions diffuse from the substrate and exert a strong inhibitory effect (remarkable at concentrations as low as 0.001 ppm) on a wide range of microorganisms such as bacteria, molds, and viruses.^38^ In combination with polyurethane matrices and polyurethane foams, silver nanoparticles showed high bactericidal activities.^44−51^ Nevertheless, the treatment of human consumer goods such as water or food with Ag^+^ ions or silver nanoparticles poses risks that need to be addressed. On the one hand, Ag^+^ ions increase their activity with increasing concentration, requiring relatively high concentrations of 0.2 ppm or more. This is the permissible value in the water consumption limit (0.2 mg L^–1^).^52^ In addition, some experiments with silver nanoparticles have shown that they can detach from parent materials and be released into the environment; aggregates of these particles are more hazardous than asbestos.^53^ For this reason, materials with an appropriate balance between their bactericidal capacity and their ability to release Ag^+^ ions or particles are preferred.

**Figure 9:**
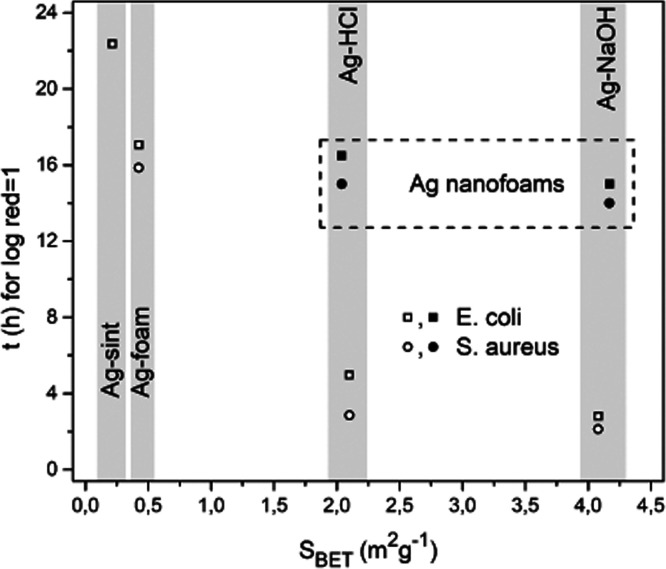
Time (h) required to reach a logarithmic reduction of 1 as a function of specific surface area (*S*_BET_ in m^2^ g^–1^) for the samples presented so far (empty symbols) and for two new samples consisting of nanoporous foams obtained by direct treatment of Al–25Ag alloy with HCl (filled circle) and NaOH (filled square).

Figure 9 shows the time required to achieve a log-reduction of 1 for the different materials. The graph includes two new materials (not yet shown for simplicity) that were evaluated under the same conditions as the antibacterial experiments. These materials correspond to the Al–25Ag alloy treated with both HCl and NaOH reagents to produce nanoporous silver foams. The specific surface area is 2.04 m^2^ g^–1^ for the HCl-treated alloy and 4.17 m^2^ g^–1^ for the NaOH-treated alloy. The graph contains information that deserves special attention. The AlAg, Ag–HCl, Ag–NaOH, and Ag-sint samples show bactericidal activity that scales with their specific surface area, such that the most active materials are Ag–NaOH and Ag–HCl, both of which exhibit a pore hierarchy. This scaling is not fulfilled in the new samples, which have developed only nanoscale porosity. Despite their specific surface area, which is comparable to those of Ag–HCl and Ag–NaOH samples, their comparatively low activity is probably due to the fact that the nanopores present in these samples do not form a network of channels with suitable dimensions to allow sufficient diffusion of Ag^+^ ions into the medium. These results are very significant as they suggest that the combination of pore sizes in hierarchical foams potentiates the diffusion of Ag^+^ ions into the medium, thereby increasing their bactericidal activity. In this context, we refer to a pore hierarchy in these foams not only in terms of size but also in terms of functionality. The smaller pores are responsible for the large specific surface area that promotes the dissolution of silver into Ag^+^ ions, and the larger pores promote the transport of these ions into the medium.

## 4 Conclusions

From the results and the discussion on the preparation and characterization of the presented materials, the following conclusions can be drawn.

- Silver foams with hierarchical porous structures can be prepared by infiltrating martyr porous preforms consisting of packed NaCl particles with Al–25Ag alloy. The infiltrated materials are immersed in water to dissolve the NaCl particles, which are considered as hard templates and lead to the formation of coarse pores. Smaller pores can be created in these foams by dealloying with acidic (HCl) or alkaline (NaOH) solutions.
- The solidification rate of the Al–25Ag alloy upon infiltration, about 5 K s^–1^, is sufficient to obtain suitable fine phase distributions to produce foams with large specific surface areas. Selective dissolution by chemical treatment with aqueous HCl or NaOH solutions gives different results. Treatment with HCl leads to complete elimination of the Al-rich α-Al(Ag) phase and aluminum in the Ag-rich β-Ag_2_Al phase, leaving Ag as the product. However, treatment with NaOH does not lead to the complete dissolution of Al, which coexists with the Ag phase after treatment as β-Ag_2_Al.
- Microstructural characterization of the materials shows that NaOH treatment leads to materials with higher densities and lower total porosity volumes than analogous materials obtained by HCl dissolution. This is mainly due to the fact that NaOH treatment cannot completely eliminate the Al content and the less dense β-Ag_2_Al phase remains. However, due to the surface texture, which strongly depends on the conditions of the treatment medium, the specific surface area generated by NaOH dealloying is higher than that obtained by HCl dealloying.
- The materials obtained by NaOH dealloying exhibit higher antibacterial activity than their HCl analogues due to their larger specific surface area. For Gram-positive (*S. aureus*) and Gram-negative (*E. coli*) bacteria, they prove to be very active materials against bacterial growth due to their 24 h bacterial logarithmic reductions of 4.5 and 4.2, respectively. Materials with pore hierarchy show much stronger antibacterial behavior than those with pores only in the nanometer range. The pore hierarchy implies a functional hierarchy in which the small pores provide a large specific surface area that facilitates the dissolution of silver into silver ions, and the coarse pores form a channel network that facilitates transport into the environment.

## References

[ref1] Lefebvre L. P.; Banhart J.; Dunand D. C. Porous Metals and Metallic Foams: Current Status and Recent Developments. Adv. Eng. Mater. 2008, 10, 775–787. 10.1002/adem.200800241.

[ref2] Ding Y.; Erlebacher J. Nanoporous Metals with Controlled Multimodal Pore Size Distribution. J. Am. Chem. Soc. 2003, 125, 7772–7773. 10.1021/ja035318g. 12822974

[ref3] Ishizaki K.; Komarneni S.; Nanko M. Porous Materials - Process Technology and Applications; Kluwer Academic Publishers, 1998.

[ref4] Hammel E. C.; Ighodaro O. L. R.; Okoli O. I. Processing and Properties of Advanced Porous Ceramics: An Application Based Review. Ceram. Int. 2014, 40, 15351–15370. 10.1016/j.ceramint.2014.06.095.

[ref5] Banhart J. Manufacture, Characterisation and Application of Cellular Metals and Metal Foams. Prog. Mater. Sci. 2001, 46, 559–632. 10.1016/S0079-6425(00)00002-5.

[ref6] Akhtar F.; Andersson L.; Ogunwumi S.; Hedin N.; Bergström L. Structuring Adsorbents and Catalysts by Processing of Porous Powders. J. Eur. Ceram. Soc. 2014, 34, 1643–1666. 10.1016/j.jeurceramsoc.2014.01.008.

[ref7] Glenk F.; Knorr T.; Schirmer M.; Gütlein S.; Etzold B. J. M. Synthesis of Microporous Carbon Foams as Catalyst Supports. Chem. Eng. Technol. 2010, 33, 698–703. 10.1002/ceat.201000005.

[ref8] Azarfar S.; Noorbakhsh F.; Salmani M.; Ansari S.; Soleymani R.; Sadighi S. In Experimental Study and Characterization of Activated Alumina Adsorbent, Proceedings of Iran International Aluminium Conference, 2016; pp 2–7.

[ref9] Erlebacher J.; Aziz M. J.; Karma A.; Dimitrov N.; Sieradzki K. Evolution of Nanoporosity in Dealloying. Nature 2001, 410, 450–453. 10.1038/35068529. 11260708

[ref10] Xiong Q.; Baychev T. G.; Jivkov A. P. Review of Pore Network Modelling of Porous Media: Experimental Characterisations, Network Constructions and Applications to Reactive Transport. J. Contam. Hydrol. 2016, 192, 101–117. 10.1016/j.jconhyd.2016.07.002. 27442725

[ref11] Song T.; Yan M.; Qian M. The Enabling Role of Dealloying in the Creation of Specific Hierarchical Porous Metal Structures—A review. Corros. Sci. 2018, 134, 78–98. 10.1016/j.corsci.2018.02.013.

[ref12] Halder A.; Patra S.; Viswanath B.; Munichandraiah N.; Ravishankar N. Porous, Catalytically Active Palladium Nanostructures by Tuning Nanoparticle Interactions in an Organic Medium. Nanoscale 2011, 3, 725–730. 10.1039/c0nr00640h. 21135970

[ref13] Huerta L.; Guillem C.; Latorre J.; Beltrán A.; Beltrán D.; Amorós P. Large Monolithic Silica-Based Macrocellular Foams with Trimodal Pore System. Chem. Commun. 2003, 1448–1449. 10.1039/b301620j. 12841285

[ref14] Asavavisithchai S.; Oonpraderm A.; Ruktanonchai U. R. The Antimicrobial Effect of Open-cell Silver Foams. J. Mater. Sci.: Mater. Med. 2010, 21, 1329–1334. 10.1007/s10856-009-3969-9. 20037777

[ref15] Ning C.; Wang X.; Li L.; Zhu Y.; Li M.; Yu P.; Zhou L.; Zhou Z.; Chen J.; Tan G.; Zhang Y.; Wang Y.; Mao C. Concentration Ranges of Antibacterial Cations for Showing the Highest Antibacterial Efficacy but the Least Cytotoxicity against Mammalian Cells: Implications for a New Antibacterial Mechanism. Chem. Res. Toxicol. 2015, 28, 1815–1822. 10.1021/acs.chemrestox.5b00258. 26258952 PMC4925100

[ref16] Goodman S. B.; Yao Z.; Keeney M.; Yang F. The Future of Biologic Coatings for Orthopaedic Implants. Biomaterials 2013, 34, 3174–3183. 10.1016/j.biomaterials.2013.01.074. 23391496 PMC3582840

[ref17] Feng Q. L.; Wu J.; Chen G. Q.; Cui F. Z.; Kim T. N.; Kim J. O. A Mechanistic Study of the Antibacterial Effect of Silver Ions on *Escherichia coli* and *Staphylococcus aureus*. J. Biomed. Mater. Res. 2000, 52, 662–668. 10.1002/1097-4636(20001215)52:4<662::AID-JBM10>3.0.CO;2-3. 11033548

[ref18] Battice D. R.; Hales M. G. A New Technology for Producing Stabilized Foams Having Antimicrobial Properties. J. Cell. Plast. 2006, 332–337. 10.1177/0021955x8502100506.

[ref19] Molina-Jordá J. M. Multi-scale Design of Novel Materials for Emerging Challenges in Active Thermal Management: Open-pore Magnesium-Diamond Composite Foams with Nano-engineered Interfaces. Composites, Part A 2018, 105, 265–273. 10.1016/j.compositesa.2017.11.020.

[ref20] Maiorano L. P.; Molina J. M. Challenging Thermal Management by Incorporation of Graphite into Aluminium Foams. Mater. Des. 2018, 158, 160–171. 10.1016/j.matdes.2018.08.026.

[ref21] Wani I. A.; Khatoon S.; Ganguly A.; Ahmed J.; Ganguli A. K.; Ahmad T. Silver Nanoparticles: Large Scale Solvothermal Synthesis and Optical Properties. Mater. Res. Bull. 2010, 45, 1033–1038. 10.1016/j.materresbull.2010.03.028.

[ref22] American Society for Testing and Materials Standard Test Method for Determining the Antimicrobial Activity of Antimicrobial Agents Under Dynamic Contact Conditions E2149-13a; ASTM Standard Book, 2013; pp 1–5.

[ref23] Fu S. W.; Lee C. C. A Study on Intermetallic Compound Formation in Ag–Al System and Evaluation of its Mechanical Properties by Micro-indentation. J. Mater. Sci.: Mater. Electron. 2018, 29, 3985–3991. 10.1007/s10854-017-8340-1.

[ref24] Yamauchi I.; Kajiwara T.; Mase T.; Saraoka M. Formation of Highly Saturated Al-Ag Precursor by Rapid Solidification for Skeletal Silver Synthesis. J. Alloys Compd. 2002, 336, 206–212. 10.1016/S0925-8388(01)01900-4.

[ref25] Zhang Z.; Wang Y.; Qi Z.; Zhang W.; Qin J.; Frenzel J. Generalized Fabrication of Nanoporous Metals (Au, Pd, Pt, Ag, and Cu) through Chemical Dealloying. J. Phys. Chem. C 2009, 113, 12629–12636. 10.1021/jp811445a.

[ref26] Song T.; Gao Y.; Zhang Z.; Zhai Q. Dealloying Behavior of Rapidly Solidified Al-Ag Alloys to Prepare Nanoporous Ag in Inorganic and Organic Acidic Media. CrystEngComm 2011, 13, 7058–7067. 10.1039/c1ce05538k.

[ref27] Wang X.; Qi Z.; Zhao C.; Wang W.; Zhang Z. Influence of Alloy Composition and Dealloying Solution on the Formation and Microstructure of Monolithic Nanoporous Silver through Chemical Dealloying of Al-Ag Alloys. J. Phys. Chem. C 2009, 113, 13139–13150. 10.1021/jp902490u.

[ref28] Basak C. B.; Sengupta A. K. Development of a FDM Based Code to Determine the 3-D Size Distribution of Homogeneously Dispersed Spherical Second Phase from Microstructure: A Case Study on Nodular Cast Iron. Scr. Mater. 2004, 51, 255–260. 10.1016/j.scriptamat.2004.04.009.

[ref29] Kim M. S.; Nishikawa H. Fabrication of Nanoporous Silver and Microstructural Change During Dealloying of Melt-spun Al-20 at.%Ag in Hydrochloric Acid. J. Mater. Sci. 2013, 48, 5645–5652. 10.1007/s10853-013-7360-3.

[ref30] Song T.; Gao Y.; Zhang Z.; Zhai Q. Influence of Magnetic Field on Dealloying of Al-25Ag Alloy and Formation of Nanoporous Ag. CrystEngComm 2012, 14, 3694–3701. 10.1039/c2ce06404a.

[ref31] Liu W.; Xin C.; Chen L.; Yan J.; Li N.; Shi S.; Zhang S. A Facile One-pot Dealloying Strategy to Synthesize Monolithic Asymmetry-patterned Nanoporous Copper Ribbons with Tunable Microstructure and Nanoporosity. RSC Adv. 2016, 6, 2662–2670. 10.1039/c5ra22978b.

[ref32] Thommes M.; Kaneko K.; Neimark A. V.; Olivier J. P.; Rodriguez-Reinoso F.; Rouquerol J.; Sing K. S. W. Physisorption of Gases, with Special Reference to the Evaluation of Surface Area and Pore Size Distribution (IUPAC Technical Report). Pure Appl. Chem. 2015, 87, 1051–1069. 10.1515/pac-2014-1117.

[ref33] Liu Z.; Yang Y.; Mi J.; Tan X.; Lv C. Dual-Templating Fabrication of Three-dimensionally Ordered Macroporous Ceria with Hierarchical Pores and its Use as a Support for Enhanced Catalytic Performance of Preferential CO Oxidation. Int. J. Hydrogen Energy 2013, 38, 4445–4455. 10.1016/j.ijhydene.2013.01.118.

[ref34] Funabashi H.; Takeuchi S.; Tsujimura S. Hierarchical Meso/macro-porous Carbon Fabricated from Dual MgO Templates for Direct Electron Transfer Enzymatic Electrodes. Sci. Rep. 2017, 7, 45147 10.1038/srep45147. 28332583 PMC5362814

[ref35] Coaty C.; Zhou H.; Liu H.; Liu P. A Scalable Synthesis Pathway to Nanoporous Metal Structures. ACS Nano 2018, 12, 432–440. 10.1021/acsnano.7b06667. 29309729

[ref36] Mai-Prochnow A.; Clauson M.; Hong J.; Murphy A. B. Gram Positive and Gram Negative Bacteria Differ in Their Sensitivity to Cold Plasma. Sci. Rep. 2016, 6, 38610 10.1038/srep38610. 27934958 PMC5146927

[ref37] Fayaz A. M.; Balaji K.; Girilal M.; Yadav R.; Kalaichelvan P. T.; Venketesan R. Biogenic Synthesis of Silver Nanoparticles and Their Synergistic Effect with Antibiotics: A Study Against Gram-positive and Gram-negative Bacteria. Nanomedicine 2010, 6, 103–109. 10.1016/j.nano.2009.04.006. 19447203

[ref38] Moerman F. Antimicrobial Materials, Coatings and Biomimetic Surfaces with Modified Microtography to Control Microbial Fouling of Product Contact Surfaces within Food Processing Equipment: Legislation, Requirements, Effectiveness and Challenges. J. Hyg. Eng. Des. 2014, 7, 8–29.

[ref39] Marambio-Jones C.; Hoek E. M. V. A Review of the Antibacterial Effects of Silver Nanomaterials and Potential Implications for Human Health and the Environment. J. Nanopart. Res. 2010, 12, 1531–1551. 10.1007/s11051-010-9900-y.

[ref40] Vimbela G. V.; Ngo S. M.; Fraze C.; Yang L.; Stout D. A. Antibacterial Properties and Toxicity from Metallic Nanomaterials. Int. J. Nanomed. 2017, 12, 3941–3965. 10.2147/IJN.S134526. PMC5449158 28579779

[ref41] Pal S.; Tak Y. K.; Song J. M. Does the Antibacterial Activity of Silver Nanoparticles Depend on the Shape of the Nanoparticle? A study of the Gram-negative Bacterium *Escherichia coli*. Appl. Environ. Microbiol. 2007, 73, 1712–1720. 10.1128/AEM.02218-06. 17261510 PMC1828795

[ref42] Ruparelia J. P.; Chatterjee A. K.; Duttagupta S. P.; Mukherji S. Strain Specificity in Antimicrobial Activity of Silver and Copper Nanoparticles. Acta Biomater. 2008, 4, 707–716. 10.1016/j.actbio.2007.11.006. 18248860

[ref43] Jung W. K.; Hye C. K.; Ki W. K.; Shin S.; So H. K.; Yong H. P. Antibacterial Activity and Mechanism of Action of the Silver Ion in *Staphylococcus aureus* and *Escherichia coli*. Appl. Environ. Microbiol. 2008, 74, 2171–2178. 10.1128/AEM.02001-07. 18245232 PMC2292600

[ref44] Ciobanu G.; Ilisei S.; Luca C. Hydroxyapatite-Silver Nanoparticles Coatings on Porous Polyurethane Scaffold. Mater. Sci. Eng., C 2014, 35, 36–42. 10.1016/j.msec.2013.10.024. 24411349

[ref45] Hsu S. H.; Tseng H. J.; Lin Y. C. The Biocompatibility and Antibacterial Properties of Waterborne Polyurethane-Silver Nanocomposites. Biomaterials 2010, 31, 6796–6808. 10.1016/j.biomaterials.2010.05.015. 20542329

[ref46] Liu H. L.; Dai S. A.; Fu K. Y.; Hsu S. H. Antibacterial Properties of Silver Nanoparticles in Three Different Sizes and their Nanocomposites with a New Waterborne Polyurethane. Int. J. Nanomed. 2010, 5, 1017–1028. 10.2147/IJN.S14572. PMC3010153 21187943

[ref47] Mulongo G.; Mbabazi J.; Nnamuyomba P.; Hak-Chol S. Water Bactericidal Properties of Nanosilver-Polyurethane Composites. Nanosci. Nanotechnol. 2012, 1, 40–42. 10.5923/j.nn.20110102.07.

[ref48] Park J. K.; Lee J. H.; Kwak J. J.; Shin H. B.; Jung H. W.; Bae S. W.; Yeo E. D.; Lee Y. K.; Yang S. S. Evaluation of an Antimicrobial Silver Foam Dressing. Wounds 2013, 25, 153–159. 25866981

[ref49] Phuong Phong N. T.; Ke Thanh N. V.; Phuong P. H. Fabrication of Antibacterial Water Filter by Coating Silver Nanoparticles on Flexible Polyurethane Foams. J. Phys.: Conf. Ser. 2009, 187, 012079 10.1088/1742-6596/187/1/012079.

[ref50] Sahuquillo Arce J. M.; Iranzo Tatay A.; Llácer Luna M.; Sanchis Boix Y.; Guitán Deltell J.; González Barberá E.; Beltrán Heras J.; Gobernado Serrano M. In Vitro Study of the Antimicrobial Properties of a Silver Ion-Releasing Polyurethane Foam. Cir. Esp. 2011, 89, 532–538. 10.1016/j.cireng.2011.02.004. 21546005

[ref51] Zeytuncu B.; Morcali M. H. Fabrication and Characterization of Antibacterial Polyurethane Acrylate-based Materials. Mater. Res. 2015, 18, 867–872. 10.1590/1516-1439.026515.

[ref52] U.S. Environmental Protection Agency. 2012 Edition of the Drinking Water Standards and Health Advisories, 2012; pp 1–20.

[ref53] Soto K. F.; Carrasco A.; Powell T. G.; Garza K. M.; Murr L. E. Comparative In Vitro Cytotoxicity Assessment of Some Manufactured Nanoparticulate Materials Characterized by Transmission Electron Microscopy. J. Nanopart. Res. 2005, 7, 145–169. 10.1007/s11051-005-3473-1.

